# Personality mediates the association between juvenile conduct problems and adulthood mood disorders

**DOI:** 10.1038/s41598-022-12939-2

**Published:** 2022-05-25

**Authors:** Jen-Hui Chan, Hsi-Chung Chen, I.-Ming Chen, Tsung-Yang Wang, Yi-Ling Chien, Shu-I. Wu, Po-Hsiu Kuo

**Affiliations:** 1 National Taiwan University Hospital Hsin-Chu Branch, Hsinchu, Taiwan; 2 Department of Psychiatry, National Taiwan University Hospital, Taipei, Taiwan; 3 Department of Medicine, Mackay Memorial Hospital, New Taipei City, Taiwan; 4 Department of Psychiatry, Mackay Memorial Hospital, Taipei, Taiwan; 5 Institute of Epidemiology and Preventive Medicine, College of Public Health, National Taiwan University, Room 501, No. 17, Xu-Zhou Road, Taipei, 100 Taiwan

**Keywords:** Psychology, Diseases, Medical research, Risk factors

## Abstract

This study aimed to examine the association between conduct problems and mood disorders, and to evaluate the mediating roles of personality traits in it. Adult participants (N = 309), for which patients with major depressive disorder (MDD) or bipolar disorder (BD), and controls without major psychiatric history were recruited. Juvenile conduct problem was defined by the items in Composite International Diagnosis Interview. We assessed personality traits of extraversion and neuroticism. Multiple mediation model was performed to investigate the intervening effect of personality traits between juvenile conduct problems and adulthood mood disorders. Participants had on average 2.7 symptoms of conduct problems, and 43.4% had conduct problems. Having more symptoms of conduct problems was associated with a higher likelihood of BD (OR = 1.20). Higher neuroticism was associated with elevated risks of both MDD and BD. There was no direct effect of binary conduct problems on the risk of BD, and showed significant total indirect effect mediated by neuroticism for BD (OR = 1.49; bias-corrected and accelerated 95% CI = 1.10–2.05), but not through extraversion. Conduct problems defined as a continuous variable had a direct effect on the risk of adult MDD (OR = 1.36; bias-corrected and accelerated 95% CI = 1.05–1.76), while had an indirect effect on the risk of BD via the mediation of neuroticism (OR = 1.08; bias-corrected and accelerated 95% CI = 1.02–1.14). Neuroticism mediates between the association of juvenile conduct problems and adult BD. This finding raises our attention to assess personality traits in individuals with juvenile conduct problems for timely intervention strategies of reducing the vulnerability for developing mood disorders.

## Introduction

Conduct disorder (CD) is among the most common psychiatric disorders in childhood and adolescence worldwide^1^. CD is characterized by a persistent and recurrent pattern of dissocial, aggressive, or defiant behaviors that violate the age-appropriate social norms and basic rights of others. Children with CD often exhibit aggressive behavior toward people and animals, destroy property, practice deceitfulness or theft, and seriously violate the rules^2^. The prevalence of conduct problems in the general population is 1.5–20%, with a male to female ratio of 3:1 to 5:1 depending on measurement method, time period, study site, and age range^3–6^. Conduct problems is related to a variety of adverse psychosocial outcomes in adulthood (crime, substance use, mental health) and creates a high family burden and significant public health expenditures^7,8^. CD in youth is suggested to increase the risk of developing other psychiatric disorders later in life. For instance, up to 30–40% of children with CD develop antisocial personality disorder in adulthood^9,10^. In addition, individuals with CD are at higher risk of mood disorders, anxiety disorders, substance abuse, and impulse control disorders throughout life^2^. A birth cohort study in New Zealand that followed 1,037 children until the age of 32 years reported that 25–60% of adults had an Axis I psychiatric diagnosis, including anxiety disorders, depressive disorders, manic episodes, substance use disorders, and a history of CD or oppositional defiant disorder^11^. Another study of more than 34,000 adults in the United States suggested that CD in childhood and adolescence was associated with Axis I and Axis II disorders, particularly substance use disorder, bipolar disorder (BD), and histrionic personality disorder^12^. Moreover, previous research reported that CD is associated with adulthood depressive and BD^13,14^. Understanding this association will help clinicians be aware of the risk of the future development of mood disorders in children and adolescents with CD.

Early studies demonstrated that specific personality traits are linked to Axis I psychiatric disorders^15,16^. Individuals with high psychoticism and neuroticism are vulnerable to mental disorders upon encountering stressful events. A survey conducted in India indicated that children with CD had higher levels of neuroticism and psychoticism than those without behavioral problems^17^. Previous studies suggested that arousal dysfunction may exist in children with CD^18^. Patients with CD might have abnormal autonomic response upon environmental stimuli, information processing deficit and maladaptive cognitive-emotional reactions^19^. Childhood is a critical stage for personality development, which derives from the interaction between temperament and environment. There was evidence that the autonomic arousal system were associated with personality traits^20–22^. Studies in children have shown that the interaction of childhood adversity with susceptibility to autonomic arousal is associated with the longitudinally-measured change in childhood personality development^23^. When the arousal response system is imbalanced, coupled with dysregulated stress response could lead to the development of neuroticism^24^. These observations lead to the postulation in the current study that childhood conduct problems would exacerbate or change towards unfavored personality types. Moreover, a study showed that high neuroticism was indicative of vulnerability to both bipolar and unipolar mood disorders^25^. It was reported that specific personality traits contribute to the development of affective disorders^26^, and affected patients seem to exhibit abnormal personality traits^27^. Therefore, personality traits might play a role in connecting juvenile and adult mental problems to some degree. With respect to mental milestone development, CD arises in childhood and personality often consolidates in early adulthood, while mood disorders begin at a later age. There is evidence of a unique relationship between childhood problems and young adult BD^28^. Whether personality traits mediate the association between juvenile conduct problems and adulthood mood disorders is unclear.

Mood disorders consist of two distinct components: BD and major depressive disorder (MDD). Prior clinical and genetic studies suggested that BD and MDD are different in genetic biomarker^29^, brain structure^30–32^, metabolic profile and immunity^33^, serum protein profile^34^, heart rate variability^35^ and treatment response^36^. Here we examined the mediating effects of personality traits between CD, BD, and MDD separately. The present study mainly focused on investigating BD while also exploring the effect for MDD with a smaller sample size. Hence, the present study aimed to utilize multiple mediation analyses to investigate the association between juvenile conduct problems and adulthood mood disorders (including MDD and BD) and the mediating role of personality traits.

## Methods

### Participants

Participants aged 20–65 years were recruited from the community and outpatient settings of six medical centers and mental health hospitals in Taiwan between October 2008 and September 2010, including healthy controls and patients with mood disorders. The patients (N = 201) were diagnosed with MDD or BD (including type I and type II) according to the Diagnostic and Statistical Manual of Mental Disorders, 4th Edition, Text Revision and not in the acute disease stage during the data collection. Eligible patients with mood disorders were referred to the study by psychiatrists. The healthy controls (n = 108) had to meet the criteria of no prior history of psychiatric illness or treatment records and no intellectual disability. They were recruited from outpatient settings in non-psychiatric departments and community settings. All eligible participants were interviewed using the Chinese version of the Composite International Diagnostic Interview (CIDI) to retrospectively collect detailed information about family history, lifetime clinical characteristics, childhood adversities, and other common psychiatric and physical disorders^37^. Any participant with a comorbidity of schizophrenia, organic brain syndrome, intellectual disability, or mood disorder secondary to substance use was excluded from the study. A total of 309 individuals were included. Extensive clinical data were acquired by trained interviewers, including sociodemographic data (age, sex, marital status, educational level, and occupational status) and mental health-related factors (alcohol use and juvenile conduct problems). Approval was obtained from the National Taiwan University Hospital Research Ethics Committee Office and Mackay Memorial Hospital Institutional Review Board. All research methods were performed in accordance with relevant guidelines and regulations. Informed consent was obtained from all participants prior to the study.

### Definition of conduct problems and mental health-related variables

This study used the 19 yes/no items of CD in the CIDI to retrospectively evaluate the juvenile symptoms of conduct disorders in the participants during adolescence. The 19 items were corresponding to 15 criteria of CD in DSM-5; 4 of them had two pertinent items in the CIDI to double-check the answers. These four criteria included ‘Stolen while confronting a victim,’ ‘Lies to obtain goods or favors or to avoid obligations, ‘Stolen items of nontrivial value without confronting a victim,’ and ‘Run away from home overnight.’ The Chinese version of CIDI was rated good in translation and in agreement with the original English version^37^. Previous study revealed that CIDI provides accurate and sensitive diagnoses for almost all nonpsychotic disorders^38^. The CIDI diagnosis has been shown to have high agreement with the majority of DSM IV diagnosis, with kappa value greater than 0.65 and excellent interrater and test-retest reliability^38,39^.

The number of conduct problems was summed over the 15 criteria in DSM-5, which ranged from 0 to 15. A dichotomous variable of conduct problems was defined as three or more positive symptoms. We also considered a more stringent definition with a cutoff of four or more symptoms based on DSM 5-TR, and performed a sensitivity analysis to evaluate the robustness of the main findings. Alcohol use was confirmed by questions in the substance use section of the CIDI and classified as no drinking, drinking without abuse, or drinking with abuse. Those who were habitual drinkers and had developed a related functional impairment were defined as alcohol abusers.

### Personality trait assessment

The Chinese version of the Eysenck Personality Questionnaire-Revised (EPQ-R) was utilized to assess major personality traits, including neuroticism, extraversion, and psychoticism^40^. In the Chinese version of the EPQ-R, the psychometric properties of extraversion and neuroticism were very good, while the internal consistency of psychoticism was low (Cronbach's alpha = 0.34). Therefore, we applied the EPQ-R to assess personality traits, extraversion, and neuroticism in the current study. There were 12 yes/no items each for extraversion and neuroticism scales. Example items were as following: ”Do other people think of you as being very lively?” for extraversion, and “Do you suffer from nerves?” for neuroticism. Scoring of extraversion and neuroticism scales was obtained by summing over the responses in each indicative item, which had a range from 0 to 12 for each trait. There were three reverse-scored items in extraversion trait. People who had high scores in extraversion are seen as social, carefree, and optimistic, while people who had high scores in neuroticism are prone to emotional distress/instability.

### Statistical analysis

All of the statistical analyses were performed using SPSS for Windows version 20. The univariate analyses were conducted using the χ^2^ test, analysis of variance, and *t*-test. Multinomial logistic regression analyses were used to examine the relationship between conduct problems and the diagnosis of mood disorders. All variables based on previous empirical and theoretical evidence were specified as covariates in the multinomial logistic regression models and multiple mediation models to control for potential confounding effects, including age, sex, marital status, education status, job, and alcohol drinking. In this study, the SPSS multiple mediation. Macro model provided by Preacher and Hayes was used to examine the intervening roles of personality traits between conduct problems and mood disorders^41^. Two individual mediation models were performed for MDD and BD, respectively. The comparison group for the specified mood disorder (i.e. MDD and BD) was healthy controls in each model. In the multiple mediation model, extraversion and neuroticism were conceptualized as two parallel intervening variables embedded in the link between conduct problems and mood disorders. Not only can the SPSS Macro model estimate the direct effect of conduct problems on mood disorders, it can estimate the indirect effects of the intervening variables. The Macro model uses a bootstrapping strategy to estimate the 95% confidence interval (CI) of the indirect effects. There was no need to assume a multivariate normal distribution when the Macro model was controlled for covariate effects. In this study, the indirect effects were bootstrapped with 5000 samples and bias-corrected accelerated (BCA) 95% CI was calculated. Statistical significance was set at *p* < 0.05.

## Results

A total of 309 participants were included in the study. Table 1 compares the sociodemographic and clinical characteristics by mood disorder diagnosis. The average participant age was 31.6 ± 7.8 years old; 42.4% of them were women. Of the participants, 56.0% were unemployed and 38.5% were habitual alcohol drinkers. Among the psychiatric diagnoses, 11.3% had MDD, 53.7% had BD, and 35.0% were healthy controls. The average extraversion score was 6.1 ± 2.6, while the average neuroticism score was 7.0 ± 3.7. The three diagnostic groups differed significantly in terms of age (*p *< 0.001); specifically, patients with BD were younger than the healthy controls (*p *< 0.001). Marital status (*p *= 0.001) and job status (*p *< 0.001) were also differentially distributed among the three groups. A total of 134 participants were with conduct problems, from 36.1% in the healthy controls to 48.2% in the BD patients (Table 1). The mean number of symptoms was ranged from 1.9 in controls to 3.1 in patients with BD. The most commonly reported symptom was “lies to obtain goods or favors or to avoid obligations,” which was seen in 47.2% of individuals. In contrast, the symptom “forced someone into sexual activity” was rarely reported in only 1% of participants (Supplementary Table 1).

**Table 1:** Comparisons of sociodemographic and clinical characteristics by psychiatric diagnoses (n = 309).

	Total (n = 309)	Psychiatric diagnoses			*p*-value for Chi-square/ANOVA
		Healthy control (n = 108)	Major depressive disorder (n = 35)	Bipolar disorder (n = 166)	
	n (%)	n (%)	n (%)	n (%)	
Age (years) (mean, SD)	31.6 (7.8)	34.0 (7.6)	32.9 (6.7)	29.8 (7.6)	*Ϝ*_(2,306)_ = 10.86, *p* < 0.001
**Sex**					
Female	131 (42.4)	47 (43.5)	20 (57.1)	64 (38.6)	χ^2^ = 4.18, *df* = 2, *p* = 0.12
Male	178 (57.6)	61 (56.5)	15 (42.9)	102 (61.4)	
**Marital status**					
Married	218 (70.6)	46 (42.6)	35 (21.1)	10 (28.6)	χ^2^ = 14.58, *df* = 2, *p* = 0.001
Separated/divorced/widowed/Single	91 (29.4)	62 (57.4)	25 (71.4)	131 (78.9)	
**Education status**					
Elementary school	12 (3.9)	3 (2.8)	1 (2.9)	8 (4.8)	χ^2^ = 1.13, *df* = 2, *p* = 0.98
Junior high school	47 (15.2)	16 (14.8)	6 (17.1)	25 (15.1)	
Senior high school	170 (55.0)	60 (55.6)	20 (57.1)	90 (54.2)	
University	80 (25.9)	29 (26.9)	8 (22.9)	43 (25.9)	
**Job**					
Unemployed	173 (56.0)	44 (40.7)	25 (71.4)	104 (62.7)	χ^2^ = 16.57, *df* = 2, *p* < 0.001
Employed	136 (44.0)	64 (59.3)	10 (28.6)	62 (37.3)	
**Alcohol drinking**					
No drinking	190 (61.5)	64 (59.3)	20 (57.1)	106 (63.9)	χ^2^ = 1.46, *df* = 2, *p* = 0.83
Drinking without abuse	73 (23.6)	28 (25.9)	8 (22.9)	37 (22.3)	
Drinking with abuse	46 (14.9)	16 (14.8)	7 (20.0)	23 (13.9)	
**Conduct problems**					
Dichotomous (≥ 3 items)	134 (43.4)	39 (36.1)	15 (42.9)	80 (48.2)	χ^2^ = 3.89, *df* = 2, *p* = 0.14
Continuous	2.7 (2.7)	1.9 (2.0)	3.0 (3.0)	3.1 (2.9)	*Ϝ*_*(*2,306)_ = 6.27, *p* = 0.002
**Eysenck Personality Questionnaire (mean, SD)**					
Extraversion	6.1 (2.6)	6.5 (2.4)	4.6 (2.4)	6.1 (2.6)	*Ϝ*_(2,306)_ = 7.70, *p* = 0.001
Neuroticism	7.0 (3.7)	4.6 (3.1)	9.0 (2.5)	8.2 (3.3)	*Ϝ*_(2,306)_ = 51.12, *p* < 0.001

Table 2 displays the univariate association between conduct problems and sociodemographic and clinical characteristics. Juvenile conduct problems were associated with male sex (*p* = 0.001), separated/divorced/widowed/single marital status (*p* = 0.02), unemployment (*p* = 0.003), and alcohol use (*p* = 0.001). Individuals with juvenile conduct problems scored higher in terms of extraversion (*p* = 0.01) and neuroticism (*p* = 0.001). Participants with conduct problems were more likely to have mood disorders (70.9%) than those without conduct problems (60.5%).

**Table 2:** Sociodemographic and clinical characteristics of participants (n = 309).

	Conduct problems (≥ 3 items)		*p*-value for Chi-square/t test
	Without (n = 175)	With (n = 134)	
	n (%)	n (%)	
Age (years) (mean, SD)	31.9 (8.0)	31.2 (7.4)	*t* = 0.79, *df* = 307, *p* = 0.43
**Sex**			
Female	88 (50.3)	43 (32.1)	χ^2^ = 10.29, *df* = 1, *p* = 0.001
Male	87 (49.7)	91 (67.9)	
**Marital status**			
Married	114 (65.1)	104 (77.6)	χ^2^ = 5.68, *df* = 1, *p* = 0.02
Separated/divorced/widowed/Single	61 (34.9)	30 (22.4)	
**Education status**			
Elementary school	7 (4.0)	5 (3.7)	χ^2^ = 1.49, *df* = 1, *p* = 069
Junior high school	23 (13.1)	24 (17.9)	
Senior high school	100 (57.1)	70 (52.2)	
University	45 (25.7)	35 (26.1)	
**Job**			
Unemployed	85 (48.6)	88 (65.7)	χ^2^ = 9.01, *df* = 1, *p* = 0.003
Employed	90 (51.4)	46 (34.3)	
**Alcohol drinking**			
No drinking	123 (70.3)	67 (50.0)	χ^2^ = 13.36, *df* = 1, *p* = 0.001
Drinking without abuse	33 (18.9)	40 (29.9)	
Drinking with abuse	19 (10.9)	27 (20.1)	
**Psychiatric diagnoses**			
Healthy control	69 (39.4)	39 (29.1)	χ^2^ = 3.89, *df* = 1, *p* = 0.14
Major depressive disorder	20 (11.4)	15 (11.2)	
Bipolar disorder	86 (49.1)	80 (59.7)	
**Eysenck Personality Questionnaire (mean, SD)**			
Extraversion	5.7 (2.6)	6.5 (2.5)	*t* = 0.62, *df* = 307, *p* = 0.01
Neuroticism	6.4 (3.8)	7.8 (3.4)	*t* = 3.25, *df* = 307, *p* = 0.001

Table 3 demonstrates the multinomial logistic regression analyses of factors associated with conduct problems and mood disorders. The presence of conduct problems was defined as two models. In model I, conduct problems were dichotomized by a cutoff of three in numbers. In model II, the total number of conduct problems was specified as a continuous variable. Conduct problems defined as a continuous variable was associated with a higher likelihood of MDD (OR, 1.12; 95% CI, 1.02–1.38) and BD diagnosis (OR, 1.20; 95% CI, 1.08–1.33). After controlling for covariates, the association with BD persisted (OR, 1.20; 95% CI, 1.08–1.33), but the association with MDD disappeared (OR, 1.17; 95% CI, 0.98–1.41). Personality traits were correlated with mood disorders. Extraversion was associated with a lower risk of MDD both in model I (OR, 0.75; 95% CI, 0.62–0.91) and model II (OR, 0.73, 95% CI, 0.60–0.88). Higher neuroticism was associated with elevated risks of MDD (OR, 1.42; 95% CI, 1.22–1.64 in model I; OR, 1.39; 95% CI, 1.19–1.61 in model II) and BD (OR, 1.34; 95% CI, 1.23–1.47 in model I; OR, 1.33; 95% CI, 1.12–1.45 in model II).

**Table 3:** Multinomial logistic regression analyses for factors associated with mood disorders.

	Major depressive disorder vs. healthy control			Bipolar disorder vs. healthy control		
		Model I	Model II		Model I	Model II
	COR (95% CI)	AOR (95% CI)	AOR (95% CI)	COR (95% CI)	AOR (95% CI)	AOR (95% CI)
Age (years)	0.98 (0.93–1.03)	1.03 (0.96–1.10)	1.03 (0.96–1.09)	0.93 (0.90–0.96)	0.96 (0.92–1.01)	0.96 (0.92–1.01)
**Sex**						
Female vs. male	1.73 (0.80–3.74)	1.78 (0.69–4.58)	2.08 (0.80–5.41)	0.81 (0.50–1.33)	0.66 (0.35–1.24)	0.73 (0.39–1.38)
**Marital status**						
Separated/divorced/widowed/ single vs. married	1.86 (0.81–4.24)	1.74 (0.61–4.99)	1.63 (0.57–4.66)	2.78 (1.63–4.73)	1.46 (0.73–2.92)	1.41 (0.70–2.83)
**Education status**						
Elementary school vs. university	1.21 (0.11–13.25)	0.33 (0.02–4.92)	0.30 (0.20–4.54)	1.80 (0.44–7.35)	2.19 (0.42–11.48)	2.01 (0.38–10.59)
Junior high school vs. university	1.36 (0.40–4.61)	0.95 (0.21–4.25)	0.92 (0.21–4.12)	1.05 (0.48–2.31)	1.38 (0.53–3.60)	1.31 (0.49–3.46)
Senior high school vs. university	1.21 (0.48–3.07)	0.78 (0.26–2.34)	0.79 (0.26–2.38)	1.01 (0.57–1.79)	1.16 (0.58–2.30)	1.18 (0.59–2.37)
**Job**						
Unemployed vs. employed	3.64 (1.59–8.32)	2.17 (0.83–5.70)	1.96 (0.75–5.15)	2.44 (1.49–4.01)	1.13 (0.62–2.08)	1.07 (0.58–1.97)
**Alcohol drinking**						
Drinking without abuse vs. no drinking	0.91 (0.36–2.32)	1.01 (0.33–3.11)	0.89 (0.29–2.75)	0.80 (0.45–1.43)	0.73 (0.35–1.52)	0.67 (0.32–1.40)
Drinking with abuse vs. no drinking	1.40 (0.51–3.88)	1.16 (0.33–4.14)	0.98 (0.28–3.49)	0.87 (0.43–1.76)	0.77 (0.31–1.92)	0.67 (0.26–1.68)
Conduct problems*						
Dichotomous (yes vs. no)	1.33 (0.61–2.88)	0.92 (0.35–2.38)	-	1.65 (1.00–2.71)	1.05 (0.57–1.95)	-
Continuous	1.12 (1.02–1.38)	-	1.17 (0.98–1.41)	1.20 (1.08–1.33)	-	1.14 (1.01–1.29)
**Eysenck Personality Questionnaire**						
Extraversion	0.72 (0.61–0.86)	0.75 (0.62–0.91)	0.73 (0.60–0.88)	0.94 (0.85–1.03)	0.97 (0.86–1.09)	0.94 (0.84–1.06)
Neuroticism	1.56 (1.33–1.82)	1.42 (1.22–1.64)	1.39 (1.19–1.61)	1.37 (1.26–1.49)	1.34 (1.23–1.47)	1.33 (1.21–1.45)

*COR* crude odds ratio, *AOR* adjusted odds ratio.

*Two definitions of presence of conduct problems were specified into the models separately. In model I, conduct problems were dichotomized by ≥ 3 and < 3 in numbers. In model II, the total numbers of conduct problems were specified into the model as a continuous variable.

**Figure 1:**
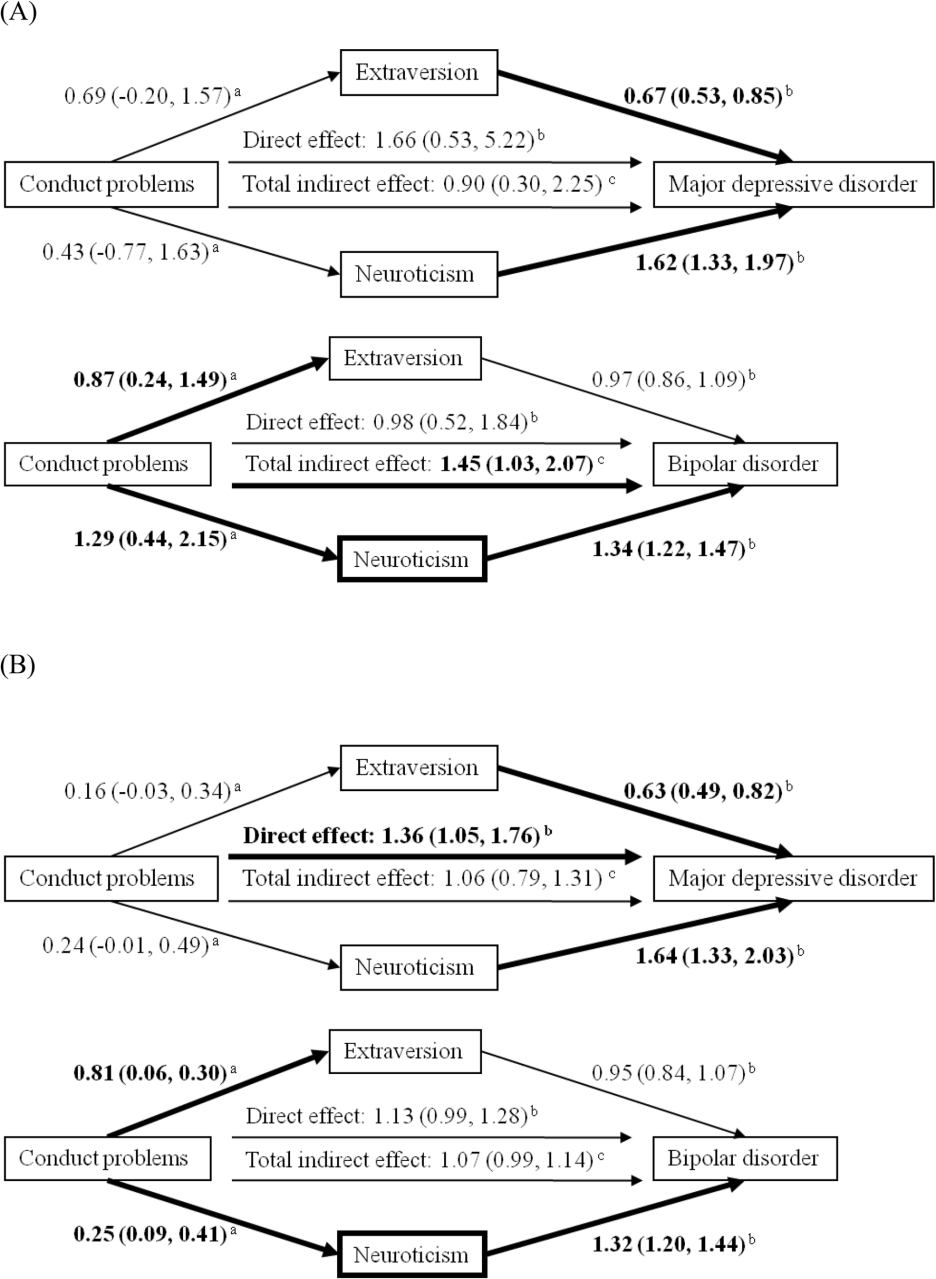
Pathways and corresponding coefficients of conduct problems correlated with mood disorders. **(A)** Effects of dichotomous conduct problems on psychiatric diagnoses mediated by extraversion and neuroticism. **(B)** Effects of total numbers of conduct problems on psychiatric diagnoses mediated by extraversion and neuroticism. ^a^Coefficients and their 95% confidence intervals on the linear regression analysis. ^b^Odds ratios and their 95% confidence intervals on the logistic regression analysis. ^c^Odds ratios and their bias-corrected and accelerated 95% confidence intervals on the logistic regression analysis. Bold lines indicated pathways with statistical significance. All models were controlled for the covariates of age, sex, marital status, education status, job, and alcohol drinking.

Figure 1 depicts the multiple mediation model analysis of the intervening effect of personality traits between conduct problems and the risk of mood disorders. In Panel A of dichotomized variable, conduct problems had neither a direct effect on the risk of adult MDD (OR, 1.66; 95% CI, 0.53–5.22) nor an indirect effect on the risk of adult MDD mediated via two personality traits (OR, 0.90; BCA 95% CI, 0.30–2.25). However, there was a total indirect effect mediated by personality traits on BD (OR, 1.45; BCA 95% CI, 1.03–2.07). The indirect effect was mainly via the path intervened by neuroticism (OR, 1.49; BCA 95% CI, 1.10–2.05) (Table 4). In Panel B of continuous variable, conduct problems had a direct effect on the risk of adult MDD (OR, 1.36; 95% CI, 1.05–1.76). In contrast, conduct problems had an indirect effect on the risk of BD via the mediation of neuroticism (OR, 1.08; BCA 95% CI, 1.02–1.14) (Table 4).

**Table 4:** Indirect effects of conduct problems on mood disorders mediated by extraversion and neuroticism.

Conduct problems	Indirect effect			
	Major depressive disorder		Bipolar disorder	
	OR	BCA 95% CI	OR	BCA 95% CI
**Dichotomous (≥ 3)**				
Total	0.90	(0.30–2.25)	1.45	(1.03–2.07)
Extraversion	0.71	(0.35–1.12)	0.97	(0.85–1.08)
Neuroticism	1.27	(0.60–2.92)	1.49	(1.10–2.05)
**Continuous**				
Total	1.06	(0.79–1.31)	1.07	(0.99–1.14)
Extraversion	0.92	(0.79–1.04)	0.99	(0.96–1.01)
Neuroticism	1.16	(0.96–1.37)	1.08	(1.02–1.14)

*BCA 95% CI* bias corrected and accelerated 95% confidence intervals.

To evaluate the impact of the stringency of the definitions of conduct problems on the main findings, we performed a sensitivity analysis by applying a cutoff of 4 (Supplementary Table 2). The results showed no intervening effects from extraversion on MDD or BD. However, neuroticism consistently intervened in the association between conduct problems and BD (OR, 1.49; BCA 95% CI, 1.09–2.09).

## Discussion

The present study included patients with mood disorders and healthy participants to explore the relationship between juvenile conduct problems, adulthood mood disorders, and personality traits. To the best of our knowledge, this is the first study to reveal the mediating roles of personality traits between juvenile conduct problems and adult BD. More precisely, neuroticism rather than extraversion intervened in the relationship between juvenile conduct problems and the risk of adult BD.

Childhood CD is reportedly part of the developmental history of adults with psychiatric disorder^11^. A 15-year longitudinal study showed that sub-syndromal CD was predictive of future BD^42^. Morcillo et al. suggested that adult BD is associated with a history of childhood and adolescent CD^12^. A systematic review of prospective studies indicated an elevated risk of BD in youth with conduct symptoms and disorders^13^. Our findings also support the association between juvenile conduct problems and adult mood disorders. The results of the multinomial logistic regression analyses showed that conduct problems as a continuous variable had a direct effect on MDD, while conduct problems as a dichotomous variable had an indirect effect on BD mediated by neuroticism.

Neuroticism is characterized by the tendency to experience frequent and intense negative emotions (anxiety, fear, frustration, guilty, emotional instability) and self-consciousness, while extraversion is characterized by sociability, assertiveness, positive emotionality, and dominance^43,44^. The relationship between personality traits and mood disorders is complex. Personality traits may predispose individuals to emanate or alter from the clinical course of mood disorders^45^. A Finnish study comparing the level of personality traits between patients with BD, MDD, and the general population found that neuroticism is an indicator of vulnerability to bi- and unipolar mood disorders^25^. A systematic review of population-based and high-risk studies of personality traits and affective disorders suggested that neuroticism was a premorbid risk factor for depressive disorder. However, previous association between personality traits and BD was less clear^46^. Since individuals with neuroticism are more sensitive to environmental stimuli, it is reasonable that neuroticism increases the risk of BD development in patients with conduct problems^24,47^.

Our results supported that symptoms of conduct problems had direct effects but not indirect effects on the risk of adult MDD. The null finding of the intervening effect of neuroticism on MDD in the present study may be partly related to the relatively small sample size. In addition, extraversion did not play an intervening role in MDD regardless of the varying definitions of conduct problems. On the other hand, juvenile conduct problems had no direct effect on BD, but there was an indirect effect mediated by neuroticism. The indirect association of conduct problems and BD mediated via the path of neuroticism suggests its statistical robustness in the clinical samples. In contrast, extraversion did not mediate the pathway between conduct problems and BD, possibly because extraverted individuals had more positive emotions and greater ability to adapt to stress and were less vulnerable to developing BD. Among personality traits, we only measured neuroticism and extraversion in this study. Openness, agreeableness and conscientiousness were not included. It was reported that low agreeableness was related to conduct problems^48^. Further study is warranted to investigate the role of agreeableness between conduct problems and adulthood mood disorders.

This finding highlights the need for increased attention to assessing personality traits in individuals with juvenile conduct problems for the early identification and timely treatment of mood disorders, particularly BD. In patients with juvenile conduct problems, guidance or counseling for personality development may lower the risk of future mood disorders.

This study had some limitations. First, the evaluation of conduct problems was retrospective and might involve recall bias. Several reviews have suggested that adults’ retrospective recalls of childhood experiences often with sufficient accuracy and can provide helpful information ^49–51^. However, we can not rule out the possibilities that individuals may have forgotten details or timing of events, denied youth conduct problems which were socially unacceptable. It’s likely to underestimate the prevalence of CD in the study and bias the results toward the null. In addition, the influence of mood symptoms on the recall accuracy maybe limited. All participants in this study were in a non-acute state. Second, conduct problems were not formally diagnosed by youth psychiatrists. Third, the precise relationship could not be inferred due to the retrospective nature of the data collection of conduct problems. We were unable to assess mood disturbances at the time of having the conduct problems. Nevertheless, according to the presumed temporal relationship between juvenile conduct problems, personality development, and mood disorder onset during development, predicting the future risk of mood disorders by recalling juvenile conduct problems and current personality traits remains reasonable and feasible. Finally, because of the relatively small sample size of participants with MDD, the absence of significant mediating effects of personality in this group requires future studies with sufficient power.

## Conclusions

Conduct problems are common childhood psychopathologies seen in the clinical setting that cause considerable family and social burdens. This study’s findings implicitly suggest that juvenile conduct problems, adult personality traits, and mood disorders may develop across the early life span and exert an impact on mood disorders through neuroticism. This finding provides us with opportunities to intervene in key contributors to mood disorders earlier in life by interrupting the chain of subsequent risk factors that may eventually lead to mood dysregulation. The mechanism underlying the indirect effect of neuroticism in patients with CD for developing adulthood mood disorders requires further investigation. More studies on psychological development are warranted to verify our findings and enable the intervention of juvenile conduct problems by addressing personality traits to improve vulnerability.

## Supplementary Information

Supplementary Tables.
