# Supplementary material for: Personality mediates the association between juvenile conduct problems and adulthood mood disorders
Source: Sci Rep. 2022 May 25;12:8866. doi: 10.1038/s41598-022-12939-2 (PMC9132998; doi:10.1038/s41598-022-12939-2)
Supplement: Supplementary file 1 — Supplementary Tables. [file 41598_2022_12939_MOESM1_ESM.docx]

| Supplementary table S1. The frequencies of individual items of conduct problems by diagnoses | | | | | |
| --- | --- | --- | --- | --- | --- |
|  | |  | Psychiatric diagnoses | | |
| Item | Description | Total (n=309) | Healthy control (n=108) | Major Depressive Disorder (n=35) | Bipolar Disorder (n=166) |
|  |  | (n, %) | (n, %) | (n, %) | (n, %) |
| **Aggression to People and Animals** | |  |  |  |  |
| 1. | Bullies, threatens, or intimidates others | 34 (11.0) | 5 (4.6) | 7 (20.0) | 22 (13.3) |
| 2. | Initiates physical fights | 49 (15.9) | 8 (7.4) | 7 (20.0) | 34 (20.5) |
| 3. | Used a weapon that can cause serious physical harm to others | 32 (10.4) | 8 (7.4) | 3 (8.6) | 21 (12.7) |
| 4. | Physically cruel to people | 26 (8.4) | 3 (2.8) | 3 (8.6) | 20 (12.0) |
| 5. | Physically cruel to animals | 32 (10.4) | 7 (6.5) | 4 (11.4) | 21 (12.7) |
| 6. | Stolen while confronting a victim | 19 (6.1) | 1 (0.9) | 3 (8.6) | 15 (9.0) |
| 7. | Forced someone into sexual activity. | 3 (1.0) | 1 (0.9) | 0 (0.0) | 2 (1.2) |
| **Destruction of Property** | |  |  |  |  |
| 8. | Deliberately engaged in fire setting | 10 (3.2) | 4(3.7) | 3 (8.6) | 3 (1.8) |
| 9. | Deliberately destroyed others’ property | 43 (13.9) | 10 (9.3) | 6 (17.1) | 27 (16.3) |
| **Deceitfulness or Theft** | |  |  |  |  |
| 10. | Broken into someone else’s house, building, or car. | 27 (8.7) | 6 (5.6) | 3 (8.6) | 18 (10.8) |
| 11. | Lies to obtain goods or favors or to avoid obligations | 146 (47.2) | 45 (41.7) | 16 (45.7) | 85 (51.2) |
| 12. | Stolen items of nontrivial value without confronting a victim | 127 (41.1) | 42 (38.9) | 12 (34.3) | 73 (44.0) |
| **Serious Violations of Rules** | |  |  |  |  |
| 13. | Stays out at night despite parental prohibitions | 110 (35.6) | 32 (29.6) | 13 (37.1) | 65 (39.2) |
| 14. | Run away from home overnight | 55 (17.8) | 8 (7.4) | 11 (31.4) | 36 (21.7) |
| 15. | Truant from school | 110 (35.6) | 29 (26.9) | 14 (40.0) | 67 (40.4) |
|  |  |  |  |  |  |
| **Cumulative items ≥ 3** | | 134 (43.4) | 39 (36.1) | 15 (42.9) | 80 (48.2) |

| Supplementary table S2. Indirect effects of conduct problems on mood disorders mediated by extraversion and neuroticism | | | | | |
| --- | --- | --- | --- | --- | --- |
|  | Indirect effect | | | | |
|  | Conduct problems ≥ 4 | | | | |
|  | Major depressive disorder | |  | Bipolar disorder | |
|  | OR | BCA 95% CI |  | OR | BCA 95% CI |
| Total | 0.81 | (0.2-2.80) |  | 1.42 | (0.99-2.07) |
| Extraversion | 0.69 | (0.34-1.15) |  | 0.96 | (0.81-1.07) |
| Neuroticism | 1.17 | (0.49-3.33) |  | 1.49 | (1.09-2.09) |
| BCA 95% CI : Bias corrected and accelerated 95% confidence intervals | | | | | |
